# Functional Epitope Core Motif of the *Anaplasma marginale* Major Surface Protein 1a and Its Incorporation onto Bioelectrodes for Antibody Detection

**DOI:** 10.1371/journal.pone.0033045

**Published:** 2012-03-12

**Authors:** Paula S. Santos, Rafael Nascimento, Luciano P. Rodrigues, Fabiana A. A. Santos, Paula C. B. Faria, João R. S. Martins, Ana G. Brito-Madurro, João M. Madurro, Luiz R. Goulart

**Affiliations:** 1 Laboratório de Nanobiotecnologia, Instituto de Genética e Bioquímica, Universidade Federal de Uberlândia, Uberlândia, Brazil; 2 Laboratório de Filmes Poliméricos e Nanotecnologia, Instituto de Química, Universidade Federal de Uberlândia, Uberlândia, Brazil; 3 Laboratório de Leishmanioses, Universidade Federal de Minas Gerais, Belo Horizonte, Brazil; 4 Laboratório de Parasitologia, Instituto de Pesquisas Veterinárias Desidério Finamor, Eldorado do Sul, Brazil; Auburn University, United States of America

## Abstract

Anaplasmosis, a persistent intraerythrocytic infection of cattle by *Anaplasma marginale*, causes severe anemia and a higher rate of abortion, resulting in significant loss to both dairy and beef industries. Clinical diagnosis is based on symptoms and confirmatory laboratory tests are required. Currently, all the diagnostic assays have been developed with whole antigens with indirect ELISA based on multiple epitopes. In a pioneer investigation we demonstrated the use of critical motifs of an epitope as biomarkers for immunosensor applications. Mimotopes of the MSP1a protein functional epitope were obtained through Phage Display after three cycles of selection of a 12-mer random peptide library against the neutralizing monoclonal antibody 15D2. Thirty-nine clones were randomly selected, sequenced, translated and aligned with the native sequence. The consensus sequence SxSSQSEASTSSQLGA was obtained, which is located in C-terminal end of the 28-aa repetitive motif of the MSP1a protein, but the alignment and sequences' variation among mimotopes allowed us to map the critical motif STSSxL within the consensus sequence. Based on these results, two peptides were chemically synthesized: one based on the critical motif (STSSQL, Am1) and the other based on the consensus sequence aligned with the native epitope (SEASTSSQLGA, Am2). Sera from 24 infected and 52 healthy animals were tested by ELISA for reactivity against Am1 and Am2, which presented sensitivities of 96% and 100%, respectively. The Am1 peptide was incorporated onto a biolectrode (graphite modified with poly-3-hydroxyphenylacetic acid) and direct serum detection was demonstrated by impedance, differential pulse voltammetry, and atomic force microscopy. The electrochemical sensor system proved to be highly effective in discriminating sera from positive and negative animals. These immunosensors were highly sensitive and selective for positive IgG, contaminants did not affect measurements, and were based on a simple, fast and reproducible electrochemical system.

## Introduction

The tick-borne intracellular pathogen *Anaplasma marginale* (Rickettsiales: Anaplasmataceae) is the causal agent of anaplasmosis, a hemoparasitic disease of cattle. *A. marginale* is distributed worldwide in tropical and subtropical regions of the world [1], resulting in considerable economic loss to both dairy and beef industries. *A. marginale* resides within erythrocytes of ruminants, and induces pyrexia, anemia, weight loss, abortion, lethargy, icterus, spleno and hepatomegaly, and often death [2]. *A. marginale* is transmitted horizontally by ixodid ticks, while mechanical transmission occurs when infected blood is transferred to susceptible cattle by fly bites or blood-contaminated fomites [3].

Six major surface proteins (MSPs) have been characterized on the erythrocytic stage of *A. marginale*. Among them, the major surface protein 1 (MSP1) has been extensively studied [4]. MSP1 is a complex of two covalently linked unrelated polypeptides, MSP1a and MSP1b. The MSP1a has been shown that is involved in the adhesion, infection and tick transmission of *A. marginale*, as well as to contribute to protective immunity in cattle [5], [6]. MSP1a contains a variable number of tandemly repeated peptides in the amino-terminal region, which are exposed extracellularly for interaction with host cell receptors [7], [8].

Many different methods have been reported for the diagnosis of *A. marginale* in cattle. The clinical diagnosis is usually based on the observation of clinical signs, necropsy findings, and the geographic region [9]. In order to confirm the diagnosis, laboratory tests such as light microscopy evaluation of Giemsa-stained blood smears or serological/molecular diagnostic procedures are required. In carrier animals, microscopy-based diagnosis can be difficult, due to variable parasitemia, and thus, a variety of serologic tests for detection of specific antibodies [10], [11] or PCR based assays [12] are necessary. However, these methods that claim high sensitivity also require greater technical skills as well as expensive instrumentation. In such a scenario, rapid identification methods using simple immunological assays for laboratory use, such as ELISA, and field portable biosensors could be more useful.

In general all antibody detection assays are based on whole antigens with multiple epitopes, which show greater sensitivity, but cross-reactions are often observed. On the other hand, epitope-specific antibody response assays are not commonly used, because it is well established that genetic background can influence the specificity of B-cell responses [13]; therefore, simple epitopes are rarely used as markers because of the difficulty in selecting common motifs that recognize broad immune responses of animals. However, the development of novel epitopes through Phage Display (PD) technology [14] has become possible, especially because selected mimotopes that mimic natural antigenic determinants are mainly originated from dominant responses, and selection favors highly reactive motifs, due to their optimized structure or functional properties [15]. Importantly, selected stable short peptide sequences assessed for tight binding to antibodies, receptors or proteins may present potential applications in diagnostics, therapeutics and vaccines [16], [17].

Because of the importance of the carrier animal in disease transmission, and also due to the difficulty in producing total purified antigens from infected erythrocyte cultures, an effective diagnostic test with synthetic peptides may be an interesting alternative tool to reduce disease transmission and economic losses. Therefore, in this present study, we have selected peptides through PD against a monoclonal antibody that targets the major surface protein 1a (MSP1a) in order to map its epitope and to develop new mimotopes that are more effective than the native epitope in detecting antibody responses in cattle against *A. marginale*. We have also proposed a bioeletrode conjugated to the epitope to detect antibodies in crude serum by electrochemistry, which may become the basis of novel biosensors based on a specific-epitope antibody response detection that can be used in field conditions due to its flexibility, easiness, fastness, and low cost.

## Results

### Epitope mapping of MSP1a by Phage Display

**Figure 1:**
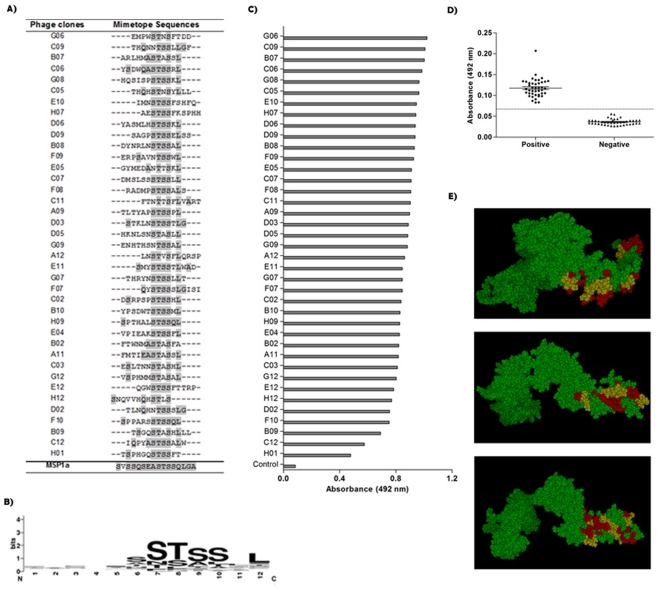
Phages selected by Phage Display and its performance. (A) Peptides sequences of 39 phage clones and their consensus sequence, according to the original sequence determined for the N-terminal region of the MSP1a protein. (B) Graphical representation of the sequence logo of MSP1a-binding motifs. The conserved sequence pattern was generated using WebLogo3 (http://weblogo.berkeley.edu/). Bits represent the relative frequency of amino acids. (C) Interaction of phage clones with the commercially available anti-MSP1 monoclonal antibody 15D2, and (D) binding specificity of each clone to pooled sera from *Anaplasma marginale* infected animals and non-infected. M13 wild type phage (without peptide) was used as a negative control. (E) Models of 3D structures predicted for the MSP1a protein and its putative epitope localization. In red, the consensus motif SxSSQSEASTSSQLGA, and in yellow, the critical motif STSSQL.

Thirty-nine randomly selected MSP1a mimotopes were obtained after three rounds of biopanning using a phage displayed 12-mer random peptide library against the anti-MSP1a monoclonal antibody 15D2 (Figure 1A). Alignment analysis revealed the consensus sequence SxSSQSEASTSSQLGA, which is depicted as a sequence logo in Fig. 1B. This sequence corresponds to tandem repeats located in the amino-terminal region of MSP1a, and may be considered part of the antigenic determinant region.

All 39 selected peptide sequences were different, but with the presence of the critical motif STSSxL in 43.6% of the clones (17/39), in which 17 of them presented the highest scores in ELISA. Alignment has also shown that 29 clones presented the full or partial sequence of the critical motif at the C-terminal end of the peptides, and all of them presented at least 4 matches with the original sequence.

Based on the frequency of residues in the selected clones and the original MSP1a sequence, we have chosen two motifs for chemical synthesis, STSSQL and SEASTSSQLGA, for additional analysis.

### Immunoreactivity of selected phagotopes for the anti-MSP1a mAb and pooled IgG from infected animals

Phage-ELISA assays were performed to validate the selected phage-fused peptide clones (phagotopes) and a successful reactivity was demonstrated for both anti-MSP1 mAb (Figure 1C) and IgG from *A. marginale* infected animals (Figure 1D). The wild type M13 phage vector (no peptide) was used as negative control to confirm the selection efficiency. The reactivities of phagotopes to the mAb were similar, except for clones C_12_ and H_01_ that presented low reactivities; however all phagotopes recognized IgG from serum of *A. marginale* infected bovines, demonstrating the ability of phagotopes to discriminate infected from non-infected animals.

To confirm the surface exposure probability of the consensus epitope sequence, we have performed a simulation to generate a 3D structure of the MSP1a protein, because its PDB structure is not available, and the putative localization of the epitope within the structure was shown in Figure 1E, corroborating the possible antibody binding region in the external sequences of the predicted protein.

### Immunoreactivity of synthetic peptides against IgG from *A. marginale* infected animals and negative controls

**Figure 2:**
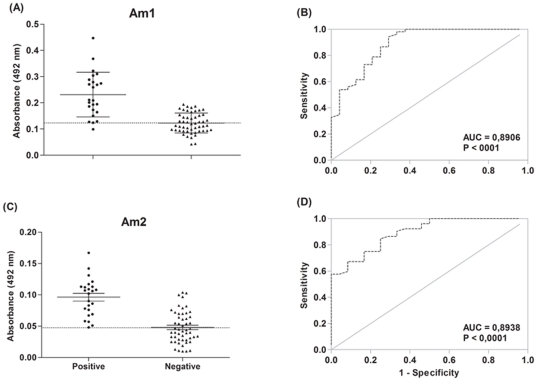
Antibody detection by ELISA. Detection of immunoglobulin G antibodies anti-*Anaplasma marginale* MSP1a in serum samples from infected and non-infected bovine with a definitive diagnosis of anaplasmosis (n = 24), and apparently healthy individuals (n = 52) by enzyme-linked immunosorbent assay using the Am1 (A) and Am2 (B), and the respectively ROC curve.

Two peptides were chemically synthesized representing the most repetitive motif (STSSQL, Am1) and the putative natural epitope (SEASTSSQLGA, Am2) based on the consensus sequence. Both synthetic molecules were able to discriminate sera from infected animals and healthy controls (p<0.0001) (Figure 2). The ROC curve analysis were significant for both peptides Am1 (AUC = 0.8906) and Am2 (AUC = 0.8938), and based on cut-off values they presented sensitivities of 95.83% and 100%, and specificities and 53.85% and 57.69%, respectively.

### Testing specificity for anaplasmosis

**Figure 3:**
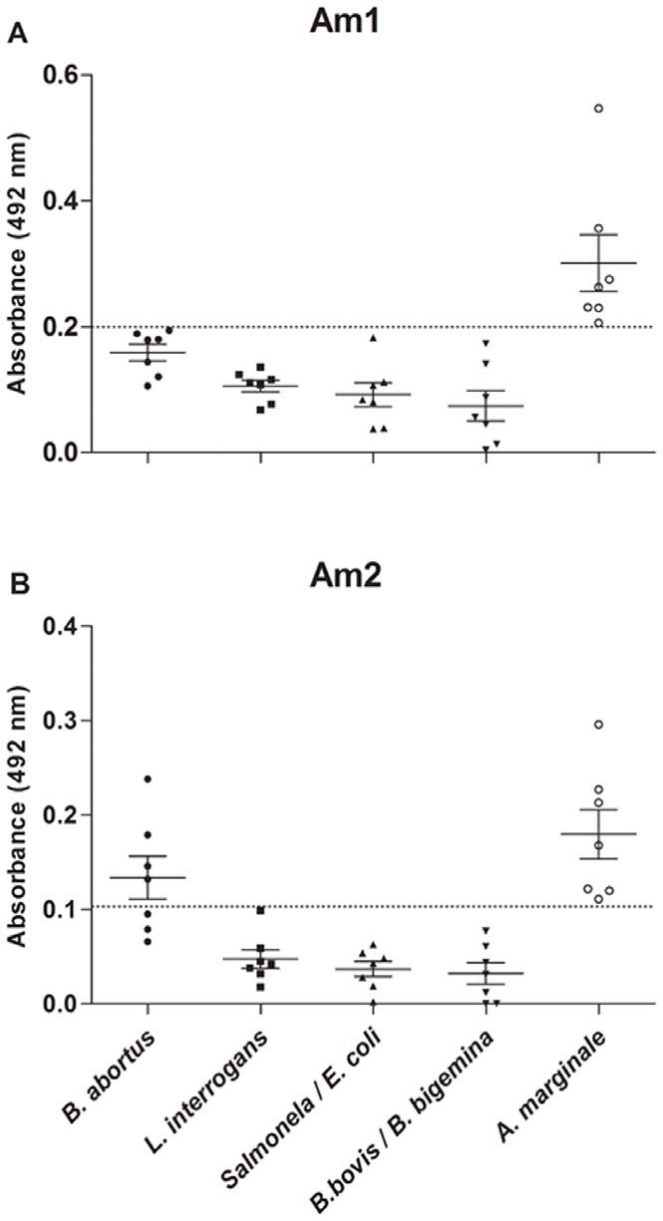
Synthetic peptides binding specificity analysis. Reactivity of synthetic peptides, Am1 (A) and Am2 (B), to bovine sera infected with *Anaplasma marginale* (n = 7), *Brucella abortus* (n = 7), *Leptospira interrogans* (n = 7), *Salmonella typhimurium* (n = 7), *Escherichia coli* (n = 7), *Babesia bovis* (n = 7), *Babesia bigemina* (n = 7); dog sera infected with *Anaplasma phagocytophilum* (n = 7), *Ehrlichia canis* (n = 7), *Ehrlichia ewingii* (n = 1); and a rabbit serum infected with *Ehrlichia sennetsu* (n = 1) by enzyme-linked immunosorbent assays.

Both synthetic peptides Am1 and Am2 presented high reactivity against sera of *A. marginale* infected animals; however, when both were tested (ELISA) for reactivity to other diseases, the Am1 specifically reacted with IgG antibodies from anaplasmosis (p<0.05), while the Am2 presented cross-reactivity with bovine brucellosis (Figure 3).

### Bioelectrode functionalization and electrochemical detection of peptide-antibody complexes

**Figure 4:**
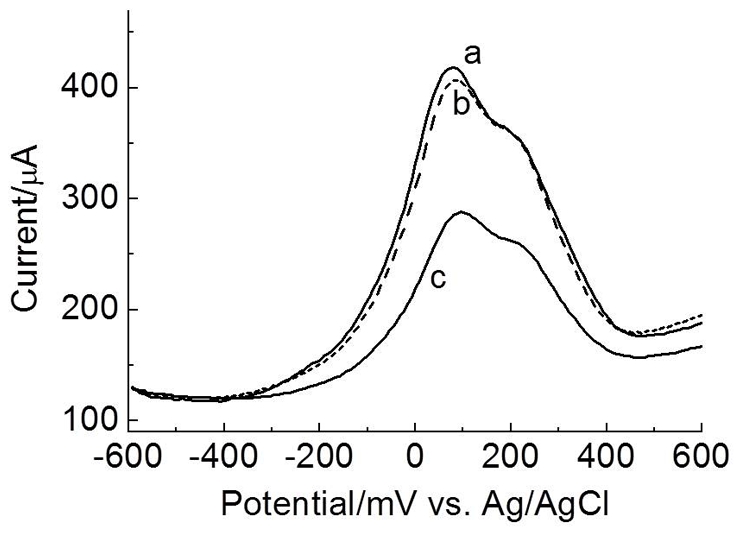
Differential pulse voltammograms of graphite electrode modified with poly(3-HPA). Synthetic peptide Am1 (A) before or after addition of negative serum (IgG−) (B) or positive serum (IgG+) (C), in aqueous solution containing K_3_Fe(CN)_6_/K_4_Fe(CN)_6_ (0.33 mmol.L^−1^) and KCl (0.1 mol.L^−1^). Modulation amplitude: 25 mV; 16 mVs^−1^.

Differential pulse voltammograms of a bioelectrode functionalized with the peptide Am1 were carried out aiming to evaluate the interaction process between the graphite electrode/poly(3-HPA)/Am1 (probe) and the target IgG (Figure 4). After immersion of the functionalized bioelectrode in a positive pooled serum sample (IgG+), it was observed a significant decrease in the amplitude of the current signal in relation to the negative serum (IgG−) with an approximate reduction of 140 µA after antibody binding.

**Figure 5:**
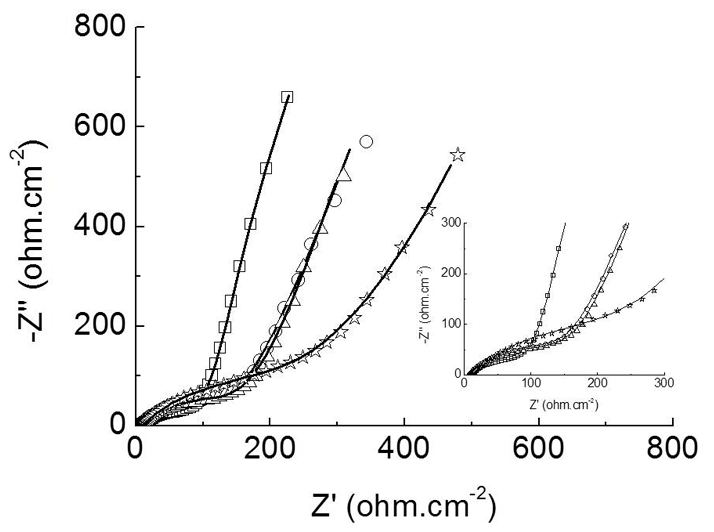
Impedance response of graphite electrode. Nyquist diagrams of a the polymeric film poly(3-HPA) (-□-), poly(3-HPA)/Am1 (-Δ-), poly(3-HPA)/Am1:IgG+ (-⋆-), and poly(3-HPA)/Am1:IgG- (-○-) obtained in aqueous solution containing K_3_Fe(CN)_6_/K_4_Fe(CN)_6_ (5 mmol.L^−1^) and KCl (0.1 mol.L^−1^), recorded from an applied potential of +0.24 V, amplitude of 10 mV, and frequency range from 100 KHz to 10 Hz. The continuous lines represent the fitting curve to the equivalent circuit. Inset: amplification of high frequencies region.

The impedance response of the graphite electrode (Figure 5) demonstrated significant changes in the surface resistivity, as shown by experimental curves for the polymeric film alone (poly(3-HPA)), the functionalized bioelectrode (poly(3-HPA)/Am1) without sera and with IgG+ and IgG− sera, generating two more curves for the bioelectrode peptide-antibody complex test. The positive serum (poly(3-HPA)/Am1:IgG+) presented a significant difference in resistivity in comparison to the three controls (poly(3-HPA)), poly(3-HPA)/Am1 and poly(3-HPA)/Am1:IgG-). The polymeric film (poly(3-HPA)) alone was different from the other two controls, which presented curves with similar behaviors.

The equivalent circuit used to fit the experimental data was: R_s_[(W R_ct,1_)Q_dl,1_]Q_dl,2_, (R_S_: solution resistance, Q_dl_: double-layer capacitance, R_ct_: charge transfer resistance, W: Warburg impedance).

Comparison of the double layer capacitance in Table 1 showed that the poly(3-HPA)/Am1:IgG+ complex presented a greater resistivity (267 Ω.cm^2^) than the controls poly(3-HPA)/Am1 (158 Ω.cm^2^) and poly(3-HPA)/Am1:IgG− (153 Ω.cm^2^). Therefore, the presence of the target positive IgG in the bioelectrode's interface presented nearly 2-fold increase in the double layer capacitance. The chi-square values (χ^2^) of the Kramers-Kronig was in the order of 10^−2^–10^−3^.

**Table 1:** Values to double layer capacitance and charge transfer resistance of the polymer-biomolecules/electrolyte interface of the bioelectrode, prepared with AC impedance analysis from Nyquist plots.

	poly(3-HPA):Am1	poly(3-HPA):Am1:(IgG+)	poly(3-HPA):Am1:(IgG-)
**Q_dl,1_**	0.018	0.033	0.013
**R_ct,1_**	158	267	153

R(Ω.cm^2^), Q(mF.cm^−2^).

**Figure 6:**
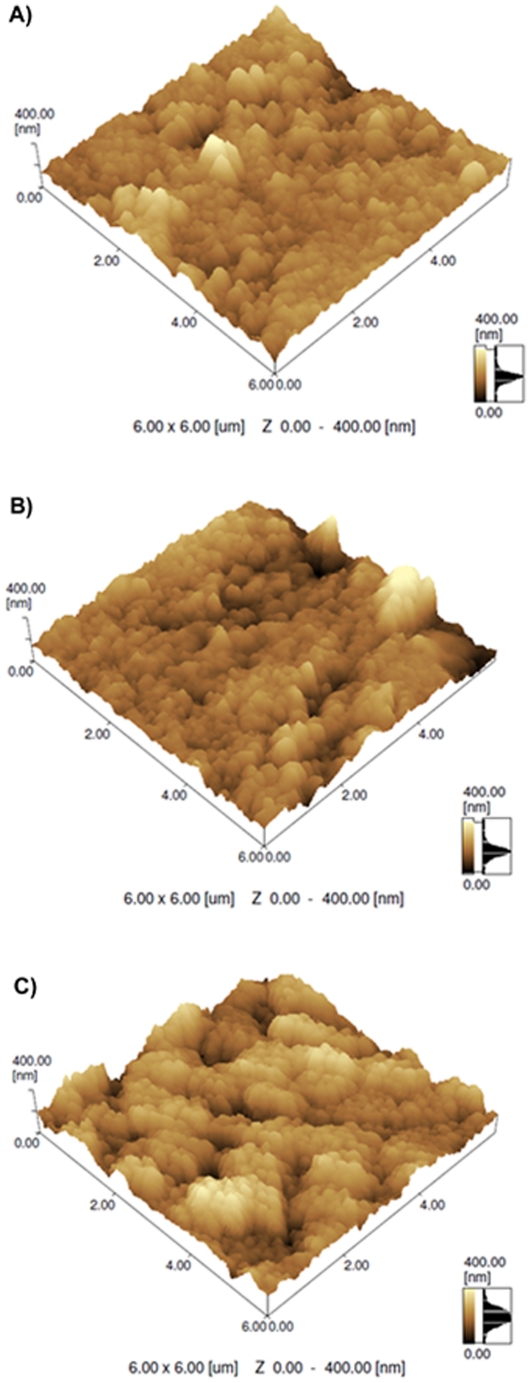
AFM topographical images of modified graphite electrode with poly(3-HPA). (A) Without biomolecules; (B) polymeric film after immobilization of the synthetic peptide Am1, and (C) agglutination process (Am1:IgG+).

The atomic force microscopic experiments (Figure 6) demonstrated that after immobilization of the Am1 (probe) on the modified electrode (Figure 6B), the bioelectrode presented a more irregular surface, but after interaction with the peptide:IgG+ (Figure 6C), the roughness of the bioelectrode increased, as observed with the formation of numerous clusters. The root-mean-square roughness values of the graphite electrode modified with poly(3-HPA), poly(3-HPA):Am1, poly(3-HPA):Am1:IgG+ were 29.7 nm, 37.0 nm and 45.9 nm, respectively.

## Discussion

In this investigation, we have used PD to select immunodominant epitopes against the neutralizing monoclonal antibody 15D2 anti-MSP1 that recognizes all geographical *A. marginale* isolates, for which two epitopes were previously characterized (QASTSS and EASTSS) [18], [19]. However, evidences have also demonstrated that the full epitope sequence consists of a larger repetitive motif of 28 or 29 amino acids (ADSSSAGGQQQESSVSSQSDQASTSSQLG) with changes in only seven residues [8]. In our mimotopes' selection of 12-mer ligands against the paratope region of the monoclonal antibody anti-MSP1a, we have revealed a shorter consensus motif (SxSSQSEASTSSQLGA) that is located within the 28-aa tandemly repeat peptide at the C-terminal side. Due to the high frequency of the critical motif at the C-terminal end, it is possible that the N-terminal end of the peptide may favor specificity and antibody binding affinity, however it is not critical due to the very large variation of residues in that region without contributing to the improved sensitivity, and apparently the alanine insertion in the critical motif (ASTSSxL) may slightly improve the reaction, although not significantly.

Interestingly, we have also demonstrated that a critical motif, STSSxL, resulted from the alignment of all selected mimotopes with the native epitope, is important for the antibody recognition and appears to play a predominant role in dictating the formation of the antigen-antibody complex and may possibly be used as a vaccine immunogen. This is corroborated by predictions of 3D structures of the MSP1a protein, regardless which model is correct, and the critical core epitope was mapped in the most exposed region of the protein in all three models, located in a hydrophilic region, and with adjacent residues in both sides showing less surface exposure, which also explain why the STSSxL was mainly selected.

This result corroborates the powerful application of the PD technology in selecting peptide ligands [20], especially against monoclonal antibodies [21], which has also proven to be an effective strategy for vaccine development [22] and drug discovery [23]. The mimotopes have strongly bound to the mAb anti-MSP1 with reactivities that were superior to the peptide that were synthetized based on the native MSP1a sequence as demonstrated by ELISA assays. Similarly, the mimotopes fused to phage particles have also recognized IgG from *A. marginale* infected bovine sera, and efficiently discriminated infected from non-infected animals. This indicates that the surface characteristics of these mimetic peptides are not only equivalent to the epitope, but PD selections may have even improved their affinity by optimizing their structure.

The final aim of this investigation was to use these short epitope peptides in bovine anaplasmosis diagnostics, but immunoassays based on single epitopes usually present low sensitivity and specificity. It is well established that the polyclonal B cell response to specific antigens is an adaptive immune system of mammals that ensures the recognition of multiple epitopes of an antigen. Because antigens can be large and complex substances, any single antibody can bind only to a single epitope, which are often specific, and highly dependent on the genetic background [13], consequently, an effective immune response frequently involves the production of many different antibodies. This means that a diagnostic tool based on a single epitope may only be effective if this antigenic determinant is highly dominant and presents a broad recognition by antibodies, which explains the rare number of publications with unique epitopes as biomarkers.

Our protein target, MSP1a, was chosen because of its occurrence in all *A. marginale* strains [18], the presence of repetitive motifs, its surface exposure, and the conserved nature across rickettsia species [19], and also because we believe that a reduced critical epitope sequence could be identified based on its dominant affinity to the antibody target, which could only be accomplished by selection and enrichment processes obtained through the PD technology.

Although peptide sequences were similar due to the critical motif STSSxL, they all differ from the native MSP1a epitope sequence, which makes them true mimotopes, but for the proof-of-principle that a minimum dominant sequence would be required for antibody recognition, we have chemically synthesized two peptides with restricted sizes based on the critical motif (STSSQL, Am1) and the consensus sequence (SEASTSSQLGA, Am2) for affinity studies. Importantly, the synthetic peptides have also presented a high reactivity against infected animal sera, which is discordant from other reports that affirm that mimotopes fused to the phage can only be detected when anchored in the phage capsid [24]–[26].

Classification of animals in the target population as infected or uninfected is conditional upon how well the reference animal population is used to validate the assay [27]. Therefore, we have used two well classified groups of animals (infected and uninfected) in order to compare with sensitivity and specificity indices reported with whole antigens [28]–[31]. The immunoassays for Am1 and Am2 synthetic peptides presented sensitivity and specificity around 96%/53% and 100%/57%, respectively. Interestingly, another study using recombinant MSP1a and MSP2 as targets reached sensitivity and specificity of 99% and 100%, respectively [32]. The differences in specificity between the epitope-based assay and the whole-antigen assay, is probably associated with other epitopes that are not recognized by the monoclonal antibody, demonstrating the differential B cell response or because short epitopes may present a higher probability of cross reactions with other proteins. However, the sensitivity remains similar, which indicates that the two restricted epitope sequences are immunodominant.

Similar to the MSP1, the whole MSP5 protein has also been successfully used for antibodies detection, with sensitivities that vary from 96 to 100% and specificities that varied from 89% to 100% [28]–[31]. Again, the slightly inferior sensitivity of Am1 compared to the whole antigens may be due to the greater number of epitopes, a hypothesis that is corroborated by another report that demonstrated the cross-reactivity of the MSP5 with multiple species of *Anaplasma* [33].

Importantly, the five additional residues in the Am2 peptide improved the sensitivity to 100%, indicating the immunodominance of the epitope, but it did not significantly differ from the assay with the critical motif (STSSxL), suggesting that this six-amino acid core epitope may have an important role in the antibody recognition. The lower specificity of ELISA tests with both synthetic peptides (Am1 and Am2) may also be due to the presence of these motifs in other pathogen species or because of cross-reactions with erythrocyte proteins demonstrated elsewhere [34].

Because of the low specificity within uninfected samples, we have also investigated cross-reactions with other ehrlichiosis (*Anaplasma phagocytophilum*, *Ehrlichia canis*, *Ehrlichia ewingii* and *Ehrlichia sennetsu*) and other bovine diseases (brucellosis, leptospirosis, salmonellosis and babesiosis), and only the Am2 peptide cross-reacted with sera of animals with brucellosis. This may suggest that the full epitope may have conformational changes in the whole antigen, which prevents the cross-reaction with other sera, but this hypothesis remains to be demonstrated, although a recombinant MSP1a has been demonstrated to be 100% specific [32]. Interestingly the critical core peptide did not react with other pathogens, which may be due to its short sequence that favors a linear recognition.

Our long-term goal is to develop peptide-based bioelectronic sensor to accurately detect animal diseases. Synthetic peptides are attractive for high precision diagnostics because they are easily produced and are free of contaminants. Therefore, once the critical core epitope was identified and validated, the next step was to incorporate this functional epitope onto bioelectrodes for electrochemical detection of infected sera without any extra manipulation and without labeling.

After functionalizing the bioelectrode with the Am1 peptide, differential pulse voltammograms were used to differentiate samples into positive and negative sera, and we have successfully demonstrated that the system was very selective for the positive sera, and although the evaluated sera contains many interfering substances, such as urea, uric acid, glutamate and albumin, none of them have disturbed the detection process.

Differently from the voltammetric sensor, an impedimetric immunosensor was also developed, which is based on the antigen–antibody complex that is formed in the interface, affecting the capacitance and the charge transfer resistance in the interface [35]. The complex plane plot, known as the Nyquist plot, representative of the impedance response of a graphite electrode, demonstrated significant electrical changes (resistance) in the presence of specific (IgG+), when the poly(3-HPA) polymer was functionalized with the Am1 peptide, which was different from the non-specific antibodies (negative sera) that were similar to the curves obtained for the polymeric film alone [poly(3-HPA)] and for the polymeric film conjugated to the peptide, confirming the high specificity of the bioelectrode. These results are compatible with our previous experiments with differential pulse voltammetry.

The model for the morphology of the electroactive layer of the bioelectrode (equivalent circuit) supports the existence of two regions: a more internal region, formed by the graphite/polymer interface, described by Q_dl,2_, and a external region, formed by the polymer-biomolecules/electrolyte interface, represented by R_ct,1_ and Q_dl,1_. The comparison of the double layer capacitance showed that the bioelectrode interface, when the peptide interacts with the specific antibody (positive sera), produced a 2-fold increase due to the electrical charges near the bioelectrode's surface. This charge transfer resistance is utilized as a main indicator in the faradaic EIS (Electrochemical impedance spectroscopy) detection for the electrode kinetics at the interface, which is modified by probes that are capable of selectively capturing a given target on the electrode's surface [36]. Comparisons of this parameter indicated that the complex poly(3-HPA)/Am1:IgG+, presented a significant increase in the R_ct_ value, resulting in a 1.7-fold decrease in the charge transfer, which was significantly different from the poly(3-HPA)/Am1 and poly(3-HPA)/peptide:IgG−. This bioelectrode corroborates other studies using impedimetric systems [37], [38].

Additional experiments with atomic force microscopy were carried out to evaluate the morphological changes in the electrode's surface, and the interaction of the peptide with the positive sera was evidenced by the formation supramolecular assemblies, with large clusters and significant conformational changes in the topographical view. The polymeric film and the conjugated film did not differ significantly from each other in the film formation, with a polymer height that varied from 100 nm to <200 nm, while for the agglutination process the range of film formation was from 200 to >300 nm, with greater roughness, which are in accordance with the voltammetric and impedimetric studies. Our results are in agreement with a report published elsewhere [39] that demonstrate that peptides obtained from PD selections can be readily used as sensing probes in biosensor development.

Interestingly, an optical immunosensor based on the anti-MSP5 antibodies detection was developed to improve diagnosis of naturally infected animals with anaplasmosis [40], but surprisingly, its sensitivity and specificity have significantly decreased (93% and 70%, respectively), probably because larger molecules may suffer important structural changes due to its interaction with polymeric surfaces.

In conclusion, we have demonstrated that highly reactive peptides selected by PD against the mAb anti-MSP1a have successfully generated the most reduced and dominant epitope motif (STSSxL) that could recognize circulating antibodies of *A. marginale* infected animals with high sensitivity, and this peptide was effectively incorporated onto a bioelectrode surface based on the polymeric film poly(3-HPA) functionalized with the critical core epitope, and both impedimetric and differential pulse voltammetric immunosensors presented a very sensitive detection of sera from *A. marginale* infected animals, resulting in a simple, fast and reproducible technique.

## Materials and Methods

### Targeted-monoclonal antibody

Monoclonal antibody against the outer membrane protein MSP1 (mAb 15D2, IgG3 isotype) used in this study was acquired Veterinary Medical Research & Development Inc., VMRD (Pullman, WA, USA).

### Peptide selection through phage display

For the peptide screening, a PhD-12 phage library (New England Biolabs, Beverly, MA, USA) was used. This is a 12-mer random peptide library fused to the minor coat protein (pIII) of the M13 bacteriophage, with a peptide diversity of 1.9×10^9^. A sample of the library containing 2×10^11^ infectious phage particles was subjected to three rounds of selection and amplification. The selection was carried out using 50 µL of Recombinant Protein G Agarose (Invitrogen) previously washed with 1 mL of TBS-T 0,1% (Tris Buffered Saline plus 0.1% of Tween 20). The Protein G Agarose was blocked with TBS-BSA 3% at 8°C for 1 h and washed four times with TBS-T 0.1%. Meanwhile, 300 ng of the monoclonal antibody anti-MSP1a was incubated with 2×10^11^ phage particles from the PhD-12 library in 200 µL of TBS-T 0,1% solution at room temperature for 20 min. Thereafter, the mAbs-phage solution was incubated with blocked agarose for 15 min at room temperature. After incubation, the resin was washed ten times with TBS-T 0,1% and the unbound phage particles discarded, followed by elution of bound phages with 1 mL of elution buffer (0.2 M Glicine-HCl, pH 2.2 and BSA 1 mg/mL) for 10 min at room temperature. After elution, the solution was centrifuged at 4000 rpm and 4°C for 1 min and the supernatant transferred to a new microtube containing 150 µL of 1 M Tris-HCl (pH 9.1) for neutralization. The eluted phages were amplified in *E. coli* ER2738 strain (New England Biolabs, Beverly, MA, USA), purified using PEG-NaCl precipitation and after each of the three rounds of biopanning, individual bacterial colonies containing amplified phage clones were grown in a microtiter plate and titrated essentially as described [15].

### Bioinformatic analysis

Phagemid DNA was isolated from 1 mL overnight cultures, and the sequencing reactions were carried out by using the DyEnamic ET Dye Terminator Cycle Sequencing Kit (GE Healthcare) with the primer −96 M13 (5′-CCCTCATTAGTTAGCGCGTAACG-3′), according to the manufacturer's instructions, and detection was performed in a MegaBace 1000 Genetic Analyzer (Amersham Biosciences) automatic capillary sequencer. Amino acid sequences were deduced according to the nucleotide sequences and analyzed using DNA2PRO2 software from Relic Program [17], [41]. The similarity of selected peptides with *A. marginale* MSP1a was performed using BLAST search followed by sequence alignment with ClustalW2 software (http://www.ebi.ac.uk/Tools/msa/clustalw2/). A graphical representation of the conserved sequence patterns within a multiple sequence alignment was generated using WebLogo3 (http://weblogo.berkeley.edu/) [42].

The three-dimensional structure predictions of the MSP1a protein were performed with the I-TASSER server [43] and the analysis was performed using PyMOL (http://www.pymol.org).

### Phage-ELISA and reactivity to the mAb 15D2

To test specific binding of these peptides to the target molecule, we performed duplicate phage-ELISA experiments. A ninety-six-well Maxisorp™ microtiter plate (NUNC, NY, USA) was coated with 1 µg/well of anti-MSP1 mAb in 50 µL of carbonate buffer (0.1 M NaHCO_3_, pH 8.6) overnight at 4°C. The wells were washed with PBS-T (phosphate-buffered saline plus 0.1% Tween 20) and then blocked for 1 h at 37°C with 3% BSA in PBS (BSA/PBS). The plate was washed twice with PBS-T and incubated with culture supernatant containing amplified phage particles (∼10^10^ pfu/mL) for 2 h at 37°C. The wells were washed four times with PBS-T followed by incubation with HRP-conjugated anti-M13 (Roche Applied Science) diluted (1∶5000) in BSA/PBS for 1 h at 37°C. The plate was washed four times in PBS-T, revealed with OPD Sigma*Fast*™ (Sigma-Aldrich) and read at 492 nm. M13 phage without displaying any peptide was used as negative control.

### Phage-ELISA with bovine serum

Serum samples from healthy and *A. marginale*-infected animals used in this study were kindly provided by The Institute Desidério Finamor of Veterinary Research (Rio Grande do Sul, Brazil).

To test the reactivity of selected clones against bovine sera from infected and non-infected animals, another phage-ELISA was carried out. Briefly, a ninety-six-well Maxisorp™ microtiter plate (NUNC, NY, USA) was coated in duplicates with phages (10^10^ pfu/mL), diluted in carbonate buffer (0.1 M NaHCO_3_, pH 8.6) overnight at 4°C. The wells were washed with PBS-T 0.05% and then blocked for 1 h at 37°C with PBS-BSA 5%. The plate was incubated for 1 h at 37°C with a pool of serum (1∶200 in PBS-BSA 5%) from animals known to be infected and non-infected with *A. marginale*. The wells were washed 3 times with PBS-T 0.05% followed by incubation with HRP-conjugated goat anti-bovine IgG (Sigma-Aldrich) diluted (1∶2000) in PBS-BSA 5% for 1 h at 37°C. The plate was washed 3 times in PBS-T 0.05%, revealed with OPD Sigma*Fast*™ (Sigma-Aldrich) and read at 492 nm. All samples were tested in duplicate. Each serum sample was tested against M13 phage without displaying any peptide as negative control.

### Peptide design and synthesis

After bioinformatics analysis of selected clones, two peptide sequences were designed and chemically synthesized by GenScript USA Inc. To increase immunogenicity, peptides were coupled to Bovine Serum Albumin. The peptide Am1 ( STSSQLGGGSSTSSQLGGGSSTSSQL ) as well the peptide Am2 ( SEASTSSQLGAGGGSSEASTSSQLGA ) were constructed with 26 residues, both containing repeats of a MSP1a motif sequence (underlined) separated by a 4-aa spacer, GGGS.

### Antibody detection by ELISA

To determine the peptides Am1 and Am2 reactivity to serum from infected and non-infected animals, specific ELISA test was carried out. High affinity microtiter plates were coated with the peptides (1 µg/weel) in carbonate bicarbonate buffer, pH 9.6, and incubated overnight at 4°C. Microplates were washed with PBS-T 0.05%. After blocking with 5% BSA in PBS at 37°C for 1 h, 100 µL/well of 24 infected and 52 non-infected bovine sera diluted in PBS-BSA 5% (1∶50 to Am1; 1∶250 to Am2) were added and incubated for 1 h at 37°C. After washing, conjugated goat anti-bovine IgG (Sigma-Aldrich) was added in a dilution of 1∶5000 in PBS-BSA and incubated for 1 h at 37°C. All samples were tested in duplicates, and the assay was developed was determined as described above.

### Specificity tests for synthetic peptides against sera from other bovine diseases

The binding specificity of synthetic peptides were analyzed by ELISA using sera from bovines infected with *Brucella abortus* (n = 7), *Leptospira interrogans* (n = 7), *Salmonella typhimurium* (n = 7), *Escherichia coli* (n = 7), *Babesia bovis* (n = 7), *Babesia bigemina* (n = 7) and *Anaplasma marginale* (n = 7); dogs infected with *Anaplasma phagocytophilum* (n = 7), *Ehrlichia canis* (n = 7), *Ehrlichia ewingii* (n = 1); and a rabbit infected with *Ehrlichia sennetsu* (n = 1), following the same protocol as previously described. Sera used in this step were kindly provided by The Institute Desidério Finamor of Veterinary Research and by the State University of Londrina.

### Construction and analysis of the bioelectrode

All reagents used were of analytical grade. Ultra high pure water (Millipore Milli-Q system) was used in the preparation of solutions. Monomer solutions, 3-hydroxyphenylacetic acid, were prepared in 0.5 mol.L^−1^ HClO_4_ solution, immediately before their use. The electrochemical experiments were conducted at room temperature (25±1°C).

The electropolymerizations were performed in three-compartment electrochemical cell connected to a potentiostat (CH Instruments, 420A-model, Austin, USA). The working electrode was graphite (99.9995%) from Alfa Aesar, in disk form, 6.18 mm of diameter. A platinum plate and electrodes of Ag/AgCl, KCl (3 M) were used as auxiliary and reference electrodes, respectively. Electrochemical impedance spectroscopy (EIS) was performed in an Autolab Electrochemical System (PGSTAT302N and FRA2 module, Eco Chemie, Utrecht, The Netherlands), using aqueous solution containing K_3_Fe(CN)_6_/K_4_Fe(CN)_6_ (5 mmol.L^−1^) and KCl (0.1 mol.L^−1^). The frequency range was from 100 KHz to 10 Hz using the open-circuit potential system, +0.24 V. The voltage amplitude was 10 mV. Film morphology and roughness values were assessed by atomic force microscopy (Shimadzu, model SPM-9600).

Graphite carbon electrodes were modified with polymer derived from 3-hydroxyphenylacetic acid [poly(3-HPA)] as described elsewhere [44].

The modified electrode with poly(3-HPA) was pre-treated by applying a potential of −0.2 V in PBS buffer, pH 7.3 for 2 minutes. After, 1 µg of synthetic peptide Am1 was diluted in the acetate buffer, pH 4.3, added on the modified electrodes and incubated for 30 min at 25°C. Graphite electrode/poly(3-HPA)/Am1 was immersed for 6 seconds in PBS buffer, pH 7.3 and dried with N_2_.

For specific *A. marginale* infected sera detection, 1 µL of positive serum in 17 µL of PBS was added to the bioelectrode (graphite electrode/poly(3-HPA)/Am1) for 15 minutes. Negative serum (1 µL) solubilized in PBS (17 µL) was used as negative control.

### Statistical analysis

Unpaired t test with Welch's correction was used to determine differences among groups for phage clones and peptides reactivity. A value of p<0.05 was considered statistically significant. Sensitivity and specificity parameters were calculated based on the ROC curve analysis. One-way analysis of variance and Tukey's Multiple Comparison test was used to determine differences among other diseases.
